# RedeAmericas: building research capacity in young leaders for sustainable growth in community mental health services in Latin America

**DOI:** 10.1017/gmh.2017.2

**Published:** 2017-02-14

**Authors:** L. Yang, C. Pratt, E. Valencia, S. Conover, R. Fernández, M. S. Burrone, M. T. Cavalcanti, G. Lovisi, G. Rojas, R. Alvarado, S. Galea, L. N. Price, E. Susser

**Affiliations:** 1 College of Global Public Health, New York University, New York, NY, USA; 2 Mailman School of Public Health, Columbia University, New York, NY, USA; 3 School of Public Health, Faculty of Medicine, University of Chile, Santiago, Chile; 4 Silberman School of Social Work, City University of New York – Hunter College, New York, NY, USA; 5 School of Public Health, National University of Córdoba, Córdoba, Argentina; 6 Institute of Psychiatry, Federal University of Rio de Janeiro, Rio de Janeiro, Brazil; 7 School of Public Health, Federal University of Rio de Janeiro, Rio de Janeiro, Brazil; 8 Clinical Hospital, Faculty of Medicine, University of Chile, Santiago, Chile; 9 School of Public Health, Boston University, Boston, MA, USA; 10 National Institute of Mental Health, National Institutes of Health, Bethesda, MD, USA; 11 New York State Psychiatric Institute, New York, NY, USA

**Keywords:** Capacity building, career development, global mental health, Latin America, mentorship program, other

## Abstract

The purpose of this paper is to describe the development and initial accomplishments of a training program of young leaders in community mental health research as part of a Latin American initiative known as RedeAmericas. RedeAmericas was one of five regional ‘Hubs’ funded by the National Institute of Mental Health (NIMH) to improve community mental health care and build mental health research capacity in low- and middle-income countries. It included investigators in six Latin American cities – Santiago, Chile; Medellín, Colombia; Rio de Janeiro, Brazil; and Córdoba, Neuquén, and Buenos Aires in Argentina – working together with a team affiliated with the Global Mental Health program at Columbia University in New York City. One component of RedeAmericas was a capacity-building effort that included an Awardee program for early career researchers in the mental health field. We review the aims of this component, how it developed, and what was learned that would be useful for future capacity-building efforts, and also comment on future prospects for maintaining this type of effort.

## Background

This paper reports on the aims and accomplishments of the capacity-building component of the RedeAmericas ‘Hub’ for mental health research. RedeAmericas was one of five interconnected regional ‘Hubs’ funded by the National Institute of Mental Health (NIMH), each of which included capacity building as a component (Schneider *et al.* in press). It brought together an interdisciplinary group of investigators from urban centers in Latin America (Argentina, Brazil, Chile, Colombia) and New York City. Although New York investigators were among the leaders who developed the initiative, RedeAmericas planned and implemented a shift toward regional leadership over the course of 5 years.

The general principles for regional capacity building in low- and middle-income countries (LMICs) were shared by all five NIMH Hubs and reflected in extensive previous work (Sharan *et al.* 2007; Sharan *et al.* 2009). In formulating the RedeAmericas capacity-building aims from these general principles, we chose to emphasize three points: (1) Research in public mental health in Latin America is growing but still very limited, partly because training programs in mental health and in public health do not prioritize public mental health. (2) Task-sharing interventions are a core element in transforming mental health services in LMICs, but there are few opportunities for training in research on task-sharing. (3) There are few self-sustaining regional networks of young researchers who can support one another as they develop. To directly address these points, three primary aims of capacity building in RedeAmericas were formulated and are reported on in this paper: (1) To select and train a cohort of ‘Awardees’ who would become independent public mental health researchers. (2) To train many (not all) Awardees to conceive, launch, nurture, supervise, and evaluate task-sharing projects. (3) To build a self-sustaining regional network of young research leaders. Barriers and facilitators for each aim were tracked and are also presented.

The Latin American context shaped the way in which we formulated these capacity-building aims. Latin America is undergoing a transition from hospital to community-based services for people with severe mental disorders. Following the 1990 PAHO Caracas Declaration (and its subsequent refinements), countries in the region made a commitment to build community-based services as alternatives to asylum care, and to add legal protections for the human rights of people with severe mental disorders (PAHO, 1990; Alarcón, 2003; Caldas de Almeida & Horvitz-Lennon, 2010; Minoletti *et al.* 2012; Alvarado *et al.* 2013). Over the past 25 years, substantial resources have been invested in community-based mental health clinics, and in efforts to increase mental health services within primary care settings (Saraceno *et al.* 2007; Rodríguez, 2010; Alvarado *et al.* 2013). Although the pace of change has been markedly uneven across countries, most have begun significant initiatives in this direction. Consequently, the next generation of mental health researchers in this region will have opportunities to develop, test, and implement innovative approaches to delivering mental health services in the community, especially for people with psychoses and other severe mental disorders.

We first describe each of the three aims identified above, how it was implemented, and what results could be measured. We provide a case illustration of an Awardee who exemplified the activities of each aim. Then we discuss what we learned about barriers and facilitators that would be useful for future capacity-building efforts. Finally, we comment on future prospects for maintaining this type of effort.

## Aims

Aim 1. To select and train a cohort of ‘Awardees’ who would become independent public mental health researchers.

Our approach to this aim included the careful selection of 14 Awardees, the development of an individually-tailored 2-year training program for each Awardee, and the monitoring of the progress of Awardees in public mental health research.

Selection of Awardees was based on their potential as early career mental health professionals who with guidance could become successful independent investigators. An application was disseminated via university departments and social networks. The selection was made by a committee comprised senior investigators and was primarily based on four criteria: previous relevant professional training, for example, a medical degree, or a research degree in an area relevant to mental health; previous publications, for example, authorship of peer-reviewed papers in regional journals; commitment to public mental health research; and the ability to read English publications. This resulted in the selection of 14 Awardees during the first 3 years.

Awardees were trained through a combination of mentorship, coursework, conference presentations, publications, grant proposals, and other activities.

### Mentorship

Mentors were local and international research experts in the area of primary interest identified by the Awardee and were expected to guide Awardees in developing plans for a research proposal. Mentors aided the Awardee in identifying a testable question, designing an appropriate research study, and if applicable, aided in data analyses and interpretation, and write-up of the study for publication. Mentors also helped foster relationships and networking among all Awardees in the program, and encouraged future collaborations.

### Coursework

Ten of the 14 Awardees participated in one or more academic or training courses in public or global mental health that were offered at various local and international universities, with associated expenses covered. The most popular were the online Epiville course offered through Columbia University (CU), summer courses at University of Chile (UCH), and the 4-week EPIC program (Epidemiology and Population Health Summer Institute) at CU. The Epiville course and EPIC program provided a basic foundation regarding epidemiologic measures, while summer courses at UCH covered a variety of mental health topics, such as human rights for people with mental disorders and qualitative methods.

Other courses Awardees attended included summer courses at King's College in London on developing community mental health services and mental health policy; an intensive epidemiology course in Lima, Peru, sponsored by the International Epidemiologic Association; a summer course at the University of North Carolina about implementation of public health interventions; and a workshop in Goa, India, on leadership in mental health.

### Conferences

Awardees attended local and international conferences relevant to their interests in mental health or epidemiologic research, again with expenses covered. Nine Awardees attended and presented at conferences around the world, including at the International Epidemiological Association Conference in Anchorage, Alaska (2014); the 2nd Global Mental Health Conference in Santiago, Chile (2015); the 1st Epidemiology Congress of the Americas in Miami, USA (2016); and other mental health and epidemiology conferences in Latin America, the USA, and Europe. Awardees also took a major role in organizing several conferences (see aim 3).

### Publications

An important indicator of progress of the training program was the peer-reviewed publications generated by Awardees. Each of the 14 career development Awardees has been an author on at least one peer-reviewed journal article either during or after their time spent in the Awardee program. Altogether, the Awardees have had 34 articles published in international journals and 63 articles in local journals. These articles are related to their work on RedeAmericas or extend from their independent research while in the Awardee program. Ten of the Awardees have been first authors on a peer-reviewed publication. Several special collections in local journals have highlighted the work of RedeAmericas and invited submissions from Awardees: Volume 72 issue 4 of the *Revista de la Facultad de Ciencias Médicas*, Córdoba, Argentina in 2015 and the *Cadernos Saúde Coletiva* in Rio de Janeiro in 2012 (issue 4) and 2013 (issue 1). These special issue articles included collaborations between Awardees, and their mentors.

### Grant proposals

Awardees took advantage of local and global funding for research focused on community mental health in Latin America. As another key indicator of progress, seven Awardees have received grants for projects during their training period. Some of these Awardees are participating in multiple grants as principal investigators and co-investigators. Overall, Awardees experienced significant success in pursuing independent research funding. Most funded grants were local, but one Awardee received a prestigious international honor – a Lisa Oehler Foundation Award – to develop research locally in Argentina with specialized training organized by CU professors. Five awardees collaborate with US and local mentors on stigma projects, including a supplement related to RedeAmericas and locally-funded projects, which serve to raise awareness throughout Latin America about mental health.

### Other activities

Six Awardees have been accepted into local university PhD programs (including the University of Córdoba and the Federal University of Rio) as well as prestigious US doctoral programs (Harvard University's leadership-focused DrPH program and Columbia University's Epidemiology PhD program) since being named as Awardees. These six Awardees understand that this opportunity was offered to them with the intention that they will use the knowledge they gain to later make contributions to community mental health initiatives in Latin America. These exceptional opportunities were partly due to the successful relationships that Awardees had with their mentors.

Adding to institutional long-term research capacity in Latin America required experiences beyond the research environment, *per se*. For instance, implementing the RedeAmericas projects and capacity-building program included negotiating different university, regulatory, and financial systems in the transfer of international subcontracts. Invaluable was the sharing between countries of locally relevant knowledge of culture and contexts, so that both scientific and contractual agendas could be finalized. During this exchange, UCH was positioned for an overall coordinating role for future international studies in the region. In line with this experience, the UCH research team led the effort in 2015 to submit a grant application to NIMH for a scale-up of the intervention that has been piloted in the RedeAmericas RCT. While the proposal was not funded, it served as a model for the process of submitting future multi-country NIMH grant proposals where UCH could take a lead role.

Finally, Awardees who received funding from local grants also received mentored training experiences in meeting human subjects’ protections standards to gain local ethical committee approvals, and in accomplishing mandatory research and administrative tasks, such as meeting timelines, submitting budgets and reports, and administering funds to recruitment sites, interviewers, and research participants.

### Awardee illustration of aim 1

We illustrate how one Awardee engaged in the above activities. Concurrent to these activities, this Awardee enrolled in a Master in Public Health program at UCH. Initially, his research interests focused on how stigma impedes recovery from mental illness. Later he broadened his interests to the use of implementation science to improve the delivery of mental health care in Latin America. His main mentorship team consisted of Rubén Alvarado (one of the two PIs in Santiago at UCH), Ezra Susser, and Lawrence Yang (US mentors).

During his Awardeeship (2013–2015), he had numerous meetings and internet conferences with his US mentors, and was also in continuous contact with Rubén Alvarado. Meetings took place during annual RedeAmericas meetings in Latin America and additional RedeAmericas meetings that took place after regional Congresses in Latin America (see below). This Awardee also made several extended visits to New York City, which helped solidify relationships with his US-based mentors. He also participated in summer courses at the EPIC program at CU in New York. He attended conferences and workshops in Santiago, New York City, Washington, D.C., Toronto, and elsewhere. Highlighting his publishing and grant-writing with mentors, which also served as important indicators of progress, he worked with Lawrence Yang to refine and adapt a cultural analytic framework for stigma. The mentorship focused on scientific writing and editing style, and several articles in peer-reviewed journals were submitted and published (Mascayano *et al.* 2015*a*; Mascayano *et al.* 2016*b*). The Awardee also applied the ‘what matters most’ framework of stigma to analyze data from a study that he conducted (Mascayano *et al.* 2015*c*). He also co-authored several additional articles on mental health service delivery in Chile (Miranda *et al*., 2013; Mascayano *et al.* 2014; Mascayano *et al.* 2016*a*).

Signifying his progression toward independent investigator status, this Awardee received a pilot grant during his second year in the program from the Chilean National Grant for Health Research and Development to conduct a pilot RCT to reduce self-stigma and to improve treatment adherence among individuals with severe mental illness (Schilling *et al.* 2015). Leading this grant provided him with valuable skills in administering grants in his local context. In addition, he applied, along with Rubén Alvarado, for other local grants, which focused on a preventive intervention for youth suicide (Mascayano *et al.* 2015*b*) and conducting a cohort study of mental illness symptoms among Chilean workers. Finally, this Awardee played a major role in the 2015 NIMH grant submission on scaling-up mental health services, described above, thus gaining significant experience in writing an NIMH grant proposal. Signifying his growing leadership role in public mental health in Chile, he was accepted in 2016 to the PhD program in Epidemiology at CU where he will continue to develop his research skills.

Aim 2. To train many (not all) of the Awardees to conceive, launch, nurture, supervise, and evaluate task-sharing projects.

Training in task-sharing projects was facilitated by the nature of the NIMH-funded regional Hubs and constituted the second major aim for Awardees. Each Hub, including RedeAmericas was required to dedicate the bulk of their funding to testing a task-sharing intervention (Collins *et al.* 2011). RedeAmericas developed and pilot-tested a task-sharing psychosocial intervention for people with severe mental disorders living in the community, Critical Time Intervention-Task Shifting (CTI-TS) (Cavalcanti *et al.* 2011; Alvarado *et al.* 2013). It was designed both to improve community-based mental health services and to extend them to a much wider population including the most disadvantaged groups. It was also designed to have a common core as well as elements that would be adaptable across the many countries in the region. The pilot testing included an RCT in two cities (Rio de Janeiro, Brazil and Santiago, Chile) and a study of implementation in a third city (Córdoba, Argentina). Most Awardees chose to participate in carrying out and evaluating the RedeAmericas CTI-TS pilot study, thereby participating in this central aim.

In CTI-TS, the task-sharing extends to both community mental health workers and ‘peer support workers’. Peer support workers have themselves experienced a severe mental disorder, and contribute special expertise to guide the recovery process; like the community mental health workers, they receive salaries for their work. Integrating peer support workers as valued experts to facilitate the recovery process was central to demonstrating that people with mental disorders can be afforded human dignity and a full societal role. This component of CTI-TS was also rather novel in Latin America, which made it both exciting and challenging, and four Awardees chose to focus their training on the peer support worker component. These Awardees were involved in identifying, selecting, and training peers in a way that respected their autonomy and potential contributions. This included participating with potential peer support workers in focus groups and helping them develop their own recovery story to share with service users. Through these training experiences, these Awardees learned to facilitate the introduction of recovery-oriented care into clinic settings. In addition, four other Awardees were active in the research but not the clinical aspect of the CTI-TS study.

### Awardee illustration of aim 2

One Awardee who has been involved in task sharing is a PhD student at the Federal University of Rio de Janeiro, Institute of Psychiatry. Her interests are in the process of recovery from severe mental disorders, and an emphasis of her PhD thesis is to examine recovery narratives for experiences of stigma. She was the Fieldwork Coordinator for the pilot RCT of CTI-TS in Rio, and has been very involved in the training of community mental health workers and peer support workers.

For this Awardee, the process of working with peers began with connections to advocacy groups and other community organizations. The project needed to be explained to these groups, and a process established for participation in several levels of training and possible selection. The trainings used a participatory methodology, including shared exercises, dialogue, and discussion. Once the intervention was underway, this Awardee worked closely with Maria Tavares Cavalcanti (her regional mentor and a Principal Investigator in Brazil of the study), in the supervision and problem solving for the intervention teams. She was also mentored by US experts in these fields.

As an Awardee, she attended research and CTI-TS trainings in New York, and relevant courses at the UCH summer program in Santiago. She attended a Leadership course designed for early career mental health professionals in Goa, India mentioned above. She has presented at regional conferences, and has two first-authored publications (Dahl *et al.* 2013; Dahl *et al.* 2015) related to her work with peers and stigma, as well as other co-authored publications.

### Aim 3. To build a self-sustaining regional network of young research leaders

The third capacity-building aim was to augment opportunities for young research leaders to learn from and plan future collaborative projects with one another, thus solidifying and adding value to their network. Several Awardees fulfilled this aim by playing a major role on the scientific organizing committees and by coordinating one or more of four important conferences (two of which are described below) that took place at RedeAmericas sites and emphasized mental health topics: the Latin American Congress of Public Health in Cordoba, Argentina (2012); the second Latin American Congress of Epidemiology in Medellin, Colombia (2014); the Conference on Stigma in Santiago, Chile (2014); and the Santiago conference on research and care for autism (2016).

The first conference described here (and further in the Awardee example below), the second Latin American Congress of Epidemiology in Medellín, took place in March 2014 with approximately 200 attendees. This was one of the first epidemiology congresses to include all of Latin America. The lead organizers were Alexandra Restrepo, who is the city PI of RedeAmericas in Medellín, along with an Awardee, who was her key partner in organizing the conference and the network that emerged from it. Attendees consisted of leading investigators and young researchers in public health from all over Latin America, including Peru, Argentina, Chile, Ecuador, Guatemala, Costa Rica, Mexico, Brazil, Bolivia, and Colombia. The Awardees who attended met with these other researchers and began to foster relationships with the goal of expanding the RedeAmericas network. Key to implementation of this aim, several of the Awardees who planned and attended these conferences have since created various social networking groups (e.g. WhatsApp, LinkedIn, and Facebook) in connection with other young researchers from various countries in Latin America (including Guatemala, Peru, Colombia, Honduras, Brazil, and Argentina) to exchange information on journals, new research, and conferences and to provide more personal support for one another.

The second conference that exemplified this aim, the Conference on Stigma in Santiago (2014), was hosted by UCH in May 2014 with over 400 attendees. One of the Awardees was the lead organizer for this conference. This event featured a seminar led by researchers from CU and Chile who had extensive experience in stigma research in vulnerable populations, and it featured presentations by Chilean and Brazilian Awardees. It was geared toward mental health professionals, service users and their families, and policymakers interested in generating discussion about mental health policies and stigma toward people with mental illness. The success of the conference was due in large part to the massive collaborative effort by the Awardees from Rio and Santiago.

Out of these conferences emerged a primary self-sustaining network, with subgroups. Multiple opportunities for collaborative grants have arisen between Awardees and their colleagues because of the pivotal relationships formed. This included collaborative grant submissions for scale-up projects of CTI-TS in Latin America. Awardees also gained knowledge by sharing experiences via their administering locally-funded projects related to mental health stigma that have been led by Awardees in several countries.

### Awardee illustration of aim 3

The Awardee in this illustration has been a leader in the development of a regional self-sustaining network. Trained as a primary care doctor in Argentina, she is currently a PhD candidate at the National University of Córdoba, with a dissertation based on a survey she conducted of mental disorders in the region. She played a significant role in the RedeAmericas team and the CTI-TS implementation led by Ruth Fernández at the University of Córdoba.

During the first year of RedeAmericas, in 2011, she and another Awardee from Santiago, as well as Alexandra Restrepo (the RedeAmericas team leader in Medellin), were accepted to attend the intensive epidemiology course offered by the International Epidemiology Association in Lima, Peru. Consequently this group of three established an ongoing connection, which has been sustained. Over time, the network was extended to include the majority of other Awardees, as well as other early career investigators across Latin America. To maintain a vibrant exchange, the network uses WhatsApp technology in addition to face-to-face meetings.

In 2012, this Awardee was a key member of the scientific organizing committee for the second Latin American Public Health Congress held in Córdoba. In 2014, she was a key organizer of the one of the first Latin American Congresses of Epidemiology, partnering with Alexandra Restrepo who led that Congress. Notably, both Congresses gave high priority to sessions on mental health. RedeAmericas meetings were held conjointly (just before or after these Congresses), maximizing the participation of Awardees as well as investigators. She attended all annual RedeAmericas meetings, and presented her work at numerous national and international conferences (including in Munich). She also made several extended visits to New York City, often alongside other Awardees. These activities made her one of the central figures in the regional network emerging from RedeAmericas, thus advancing this third aim.

## Barriers and Facilitators

### Aim 1

Several prominent barriers and facilitators emerged in the implementation of the Awardee program. One barrier, likely not unique to RedeAmericas, was in the establishment of contractual relationships between institutions of different countries. At times it was challenging for the various financial administrations to work together to meet each other's differing requirements, as mentioned above. Although this challenge did, on a number of occasions, cause delays in the transfer of funds, all are now better equipped to work together in the future.

On an anonymous survey administered at the end of the Awardee program, nearly all Awardees reported being satisfied with their local mentor, with whom they worked closely on their career development. Major facilitators to the successful relationships with their local mentors included having a pre-existing relationship with the mentor, the ease of communicating in the local language, and being able to schedule periodic face-to-face meetings. Some Awardees forged a strong, productive relationship with their US mentors, too, while others found this relationship to be the weaker of the two. While we do not have specific reasons for this, we speculate that barriers to this approach were language or cultural differences that presented an obstacle to communication, and fewer opportunities to meet face-to-face. For some Awardees, annual meetings of RedeAmericas in Latin America facilitated face-to-face meetings with mentors. Throughout the program, many of the US mentors traveled to RedeAmericas sites for research, or to teach classes or workshops at the mentees’ affiliated universities on mental health-related topics, which also provided valuable meeting opportunities.

### Aim 2

Several notable barriers and facilitators emerged in the training of Awardees who participated in the peer-based task-sharing interventions. One barrier was that Awardees and their mentors were often not yet familiar with implementing a peer-based approach; partnering with peers in recovery is relatively novel to Latin America. Overcoming this required facilitating discussion, reviewing empirical evidence, and most importantly, experiential learning to witness the key roles that peer support workers played in role-modeling recovery for service users. Another barrier was the sometimes negative attitudes of services toward peers being paid to participate in care, as clinics and unions were initially reluctant to offer financial pay. An associated barrier was contending with unions and pay scales when peers were provided financial compensation for their services, which varied across each locale. Setting up payments thus required systems-level innovations in reimbursement procedures. A final barrier was that more connections were needed to encourage development of task sharing capacities for the Awardees who were not personally involved in implementing the RedeAmericas task sharing intervention. Key facilitators included Awardees being placed in mental health clinics where peer support worker recovery services were being implemented; this opened the perspective of the Awardees and clinical staff to the beneficial role that peer support workers had in encouraging recovery. As the RedeAmericas project has progressed, CTI-TS has ultimately been much more accepted than first expected, which augurs further implementation of this task-sharing intervention in other regions of Latin America.

### Aim 3

A barrier to sustaining this regional network of Awardees after award funds are depleted is the cost of travel to meet face-to-face, which was essential for forming productive relationships. Another potential barrier is the time, effort, and financial support required to organize conferences; furthermore, it is difficult to free enough time from other professional activities to attend each meeting. Key facilitators included a strong motivation to learn from peers who were advancing similar work in other countries, partly deriving from a desire to learn from each other, and partly deriving from the overarching political context that emphasized human rights among Latin American countries in this period. Another facilitator was that the Awardees were provided multiple opportunities to meet each year, whether it was at a conference, annual RedeAmericas meetings, or in the USA, which greatly fostered enduring relationships. Finally, the use of technology, such as the WhatsApp network, offered convenient platforms for ongoing interaction among Awardees.

## Conclusion

The capacity-building program described herein was a core aspect of a broader RedeAmericas initiative. To implement aim 1, we chose to focus on 14 carefully selected Awardees who could be trained at a high level. All were trained in mental health research relevant to improving mental health services in Latin America, and the vast majority produced publications and grants, and/or proceeded to obtain higher degrees, demonstrating progress toward becoming independent investigators. To achieve aim 2, most Awardees also learned about the development and evaluation of task-sharing interventions, often through direct participation in the RedeAmericas trial. To fulfill aim 3, many Awardees became a driving force behind a regional network of early career investigators in Latin America who exchange ideas among one another and have played key roles in organizing conferences and other activities. Overall, therefore, we believe that these three aims were achieved and could make an important contribution to transforming mental health services in the region. The barriers and facilitators that we encountered have been described for each aim and will be informative for future efforts of this kind.

Finally, we emphasize two ongoing ‘cross-cutting’ challenges because of their central import for the future of this effort as well as for subsequent efforts along these lines. Although these two challenges were briefly noted under the specific aim to which they most directly pertain, their effects actually cut across all aims. One pertains to the administration and disposition of funds across numerous institutions in different countries with their own regulations, in tandem with accountability to NIMH. Language barriers add another layer of complexity to these administrative challenges. Official documents within and between Latin American countries need to be in Spanish or Portuguese (and sometimes both), while official documents for NIMH and other US institutions need to be in English. This challenge proved to be greater than anticipated, and despite substantial progress, overcoming it is still a work in progress. We propose that training in the administrative arena and in the parallel use of multilingual documents could be built into future efforts that are funded from outside Latin America.

The second pertains to building a regional network of young investigators that can sustain itself over a long period as required to ‘transform the future’. We were more successful than anticipated in building a strong network over the course of 5 years, but now need to ensure that it continues to flourish and expand, rather than gradually fading after the grant funding ends. The young investigators themselves, with the support of their mentors, have been concerned about sustaining their relationships and are actively engaged in efforts to do so. It is, however, difficult for them to do so without funding specifically targeted for that purpose. For example, we learned that even though much communication could occur electronically, face-to-face meetings had a catalytic effect and were an essential element to building and sustaining long-term relationships. We have secured some support for the year after the end of the grant, that will help support face-to-face meetings, and extend the network of young investigators to selected other countries in Latin America (e.g. Peru and Bolivia), but this will not be sufficient for optimal results. Based on our experience in RedeAmericas, we suggest that ten rather than 5 years of support is needed to solidify this kind of network as a base for the future. Although only a small amount of funding would be needed, we anticipate that it will be difficult to obtain regionally-focused funding in a single Latin American country. Therefore, it will be important for NIMH and other institutions with a global remit to dedicate a small amount of funding to sustain networks built by the ‘Hubs’ (or other equivalent networks) until they are truly self-sufficient.
